# Modeling Effects of Vertebrate Host Exclosures and Host-Targeted Acaricides on Lone Star Tick (*Amblyomma americanum*, L.) Infestations

**DOI:** 10.3390/pathogens11121412

**Published:** 2022-11-24

**Authors:** Hsiao-Hsuan Wang, William E. Grant, Taylor G. Donaldson, Pete D. Teel

**Affiliations:** 1 Ecological Systems Laboratory, Department of Ecology and Conservation Biology, Texas A&M University, College Station, TX 77843, USA; 2 Department of Entomology, Texas A&M AgriLife Research, College Station, TX 77843, USA

**Keywords:** disease vectors, tick population dynamics, spatial-temporal dynamics, simulation models, tick control

## Abstract

We used a spatially explicit model to simulate the potential effects of exclosures and acaricides targeted at medium-sized mammalian hosts on the local distribution and abundance of lone star ticks (*Amblyomma americanum*) within forestlands of the southeastern United States. Both exclosures and acaricides were successful in markedly reducing the densities of all off-host tick life stages inside the treatment areas. Densities dropped to almost zero immediately inside the edges of the exclosures, with noticeably depressed densities extending outward 30 to 60 m from the exclosures, and the simulated exclosures maintained their effectiveness as their sizes were decreased from 4.5 to 2.25 to 0.8 ha. Densities exhibited a smooth gradient across the edges of the acaricide-treated areas, with depressed densities extending ≈100 m outward from the edges, but with perceptible densities extending ≈60 m inward from the edges; thus, the simulated acaricide areas lost their effectiveness as size was decreased to slightly less than one-half the diameter of the activity range of the targeted host. Our simulation results indicated that off-host nymph densities responded to reductions of medium-sized host densities. These results suggest that targeting acaricides at medium-sized hosts may be an effective, and currently under-utilized, method for tick suppression.

## 1. Introduction

The lone star tick (*Amblyomma americanum* (L.)) is found throughout the southeastern United States, utilizes a wide range of hosts including humans [1], and has been identified as a vector of public health significance in the United States [2,3]. The lone star tick is a potential vector of various pathogens such as *Rickettsia rickettsi* (Rocky Mountain spotted fever) [4,5], *Pasteurella tularensis* (tularemia) [4], *Coxiella burneti* (Q fever) [6], *Borrelia burgdorferi* (Lyme disease) [7], *Borrelia lonestari* [8], and *Ehrlichia* spp. [9,10]. Attempts to limit the spread of tick-borne pathogens usually involve tick control [11]. For example, the Northeast Area-wide Tick Control Project funded by the U.S. Department of Agriculture used acaricide-treated 4-Poster Deer Treatment Bait Stations in five eastern states to control ticks feeding on white-tailed deer (*Odocoileus virginianus*) from 1997 to 2002 [12]. Alternatives to the application of area-wide acaricides, which may be unacceptable to nearby residential areas [13], include reduction of host densities [14,15], exclusion of definitive hosts [2,3,16,17,18,19], host-targeted acaricides [13,20], habitat modification, and controlled burns [21]. A prerequisite for improving the efficacy of tick control is an understanding of the processes involved in host–parasite–landscape interactions at local scales under a wide range of conditions [22,23]. Such an understanding is difficult to attain by means of empirical observations alone. Over the past two decades, numerous models of tick population dynamics have been developed [24,25,26]. However, only recently have models been able to provide sufficient spatial-temporal detail needed to represent the potential effects of local and/or periodic tick control measures [27,28,29,30,31], such as the use of host exclosures and host-targeted acaricides.

Exclosures usually are targeted at deer while acaricides are usually applied to deer feeder stations or to rodent bait boxes [13,20]. However, field studies have indicated that medium-sized hosts contribute significantly to maintaining populations of blood-fed larval and nymphal ticks [32], and results of simulations conducted by Wang et al. [33,34,35] have indicated that off-host nymph densities are affected noticeably by reductions in the densities of medium-sized hosts. This suggests that medium-sized hosts may represent an effective host target. However, currently, no acaricides are approved nor delivery technologies proven for application to medium-sized hosts, and we are unaware of field experiments involving the exclusion of medium-sized hosts. In this study, we use a simulation model to explore the potential effects of (1) the physical exclusion of medium-sized hosts and (2) the application of acaricides to medium-sized hosts. We simulate these hypothetical scenarios within the context of lone star tick population dynamics within forestlands of the southeastern United States.

## 2. Materials and Methods

**Figure 1:**
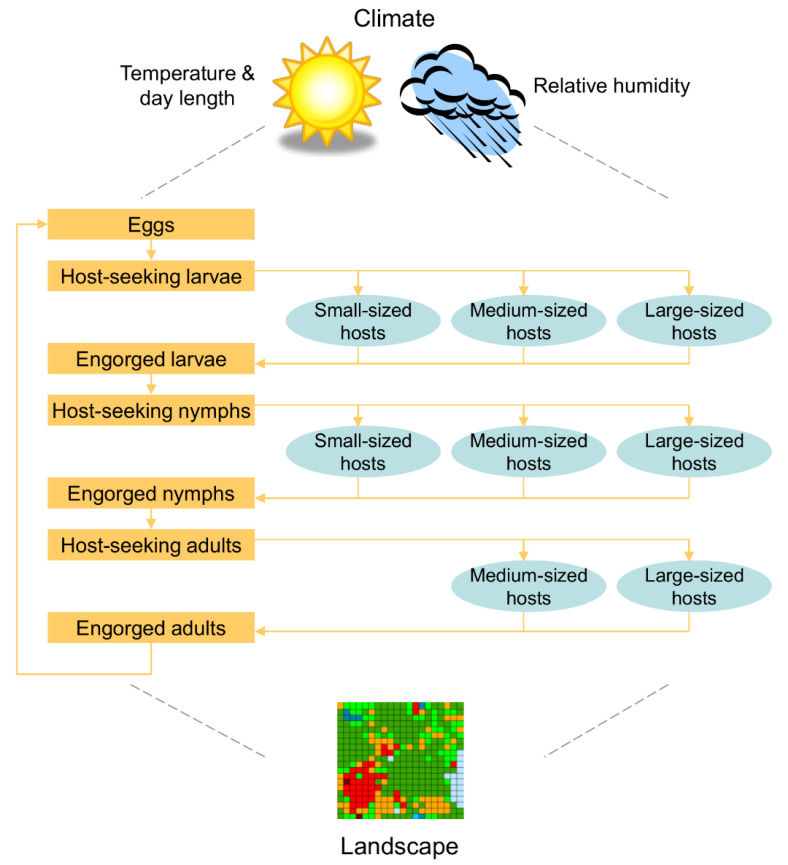
Conceptualization of the model used in this study. Yellow boxes represent life stages of the lone star tick [36], blue ovals represent alternative hosts, and yellow lines indicate alternative pathways that can be used to obtain the three blood meals needed to progress through the life stages Adapted from Wang et al. (2012) [33].

**Figure 2:**
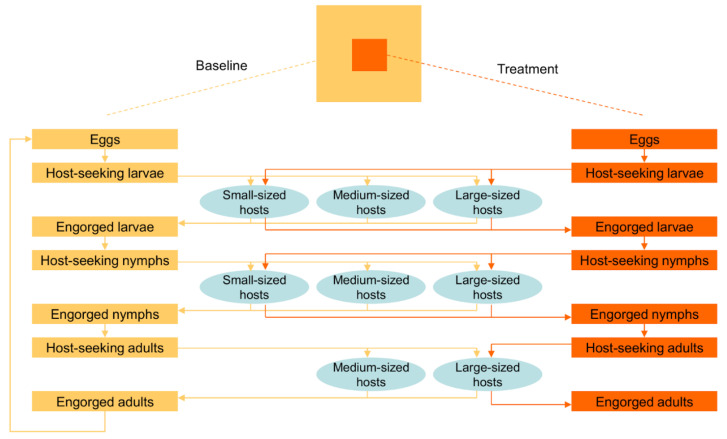
Conceptualization of the effects of the physical exclusion of medium-sized hosts and the application of acaricides to medium-sized hosts on the various blood meal pathways illustrated in Figure 1. Ticks within the treatment area (orange area) can obtain blood meals only from small- and large-sized hosts (indicated by orange lines), whereas ticks outside the treatment area (yellow baseline area) can obtain blood meals from host of all three sizes (indicated by yellow lines).

We used the spatially explicit model developed by Wang et al. [33] (Figure 1) to represent (1) the physical exclusion of medium-sized hosts and (2) the application of a hypothetical acaricide to medium-sized hosts (Figure 2). The model is a spatially structured, individual-based, stochastic model consisting of a square lattice of 400 cells, each representing a 30 m by 30 m (0.09 ha) habitat patch within a (≈40 ha) simulated landscape [33]. A detailed model description is available in Wang et al. [33].

**Figure 3:**
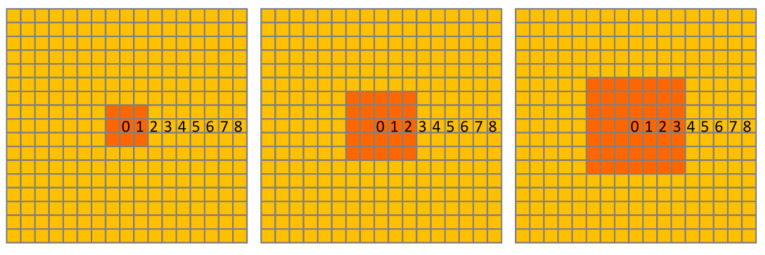
Design of an approximately 0.8 ha (9 cells), 2.25 ha (25 cells), or 4.5 ha (49 cells) treatment area (orange) within the baseline area (yellow). Numbers present the distance (number of cell) from the central grid cell.

We simulated three scenarios in which medium-sized mammalian hosts were excluded from an approximately 0.8 ha (9 cells), 2.25 ha (25 cells), or 4.5 ha (49 cells) area, and three scenarios in which acaricides were applied to all medium-sized hosts when they were within an approximately 0.8 ha, 2.25 ha, or 4.5 ha area from the central grid cell (Figure 3). Medium-sized mammalian hosts are those whose body weight is between 2 and 15 kg in southeast Texas including nine-banded armadillo (*Dasypus novemcinctus*), swamp rabbit (*Sylvilagus aquaticus*), black-tailed Jackrabbit (*Lepus californicus*), nutria (*Myocastor coypus*), red fox (*Vulpes vulpes*), gray fox (*Urocyon cinereoargenteus*), raccoon (*Procyon lotor*), American badger (*Taxidea taxus*), striped skunk (*Mephitis mephitis*), river otter (*Lontra canadensis*), and Bobcat (*Lynx rufus*) [37]. The list of small- and large-sized mammalian hosts is available in Wang et al. [33]. We assumed that all the targeted hosts, and only the targeted hosts, were excluded from the treatment areas, and that acaricides killed all ticks that attach to the targeted host within one week. All simulations and scenario treatments lasted four years, and we summarized model output in terms of peak densities of off-host larvae, nymphs, and adults during the late-summer/early-fall (≈week 35 for adults and ≈week 40 for larvae and nymphs) at different distances from the center of the treatment area during the 4th (last) year of simulated time.

## 3. Results

**Figure 4:**
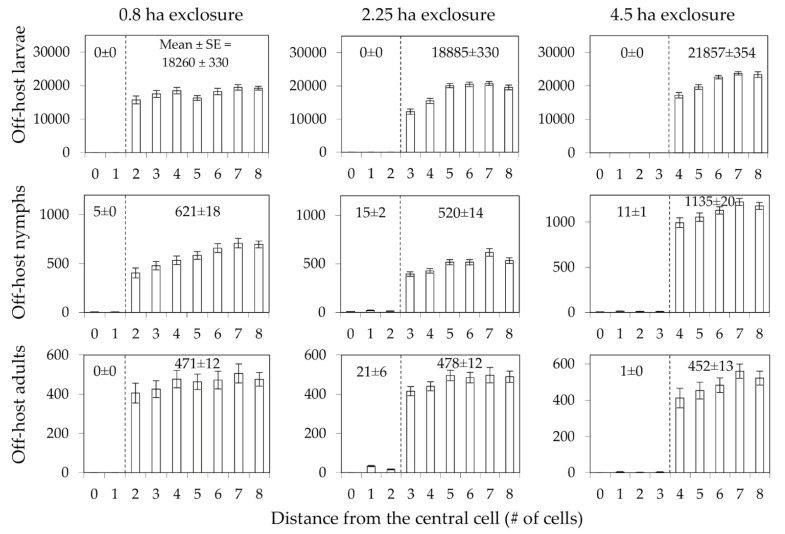
Densities of off-host ticks (larvae, nymphs, and adults; individuals/ha) at the indicated distances from the center of 4.5, 2.25, and 0.8 ha exclosures that prevented entry of medium-sized hosts. Bars represent means and standard errors of late-summer/early-fall peak densities (≈week 35 for adults and ≈week 40 for larvae and nymphs) during the 4th (last) year of simulated time.

All six scenarios were successful in markedly reducing the densities of all off-host tick life stages inside the treatment areas. Densities dropped to almost zero immediately inside the edges of the exclosures, with noticeably depressed densities extending outward 30 to 60 m from the exclosures (Figure 4). Thus, the simulated exclosures maintained their effectiveness in drastically reducing local tick densities as the size of the exclosure was decreased.

**Figure 5:**
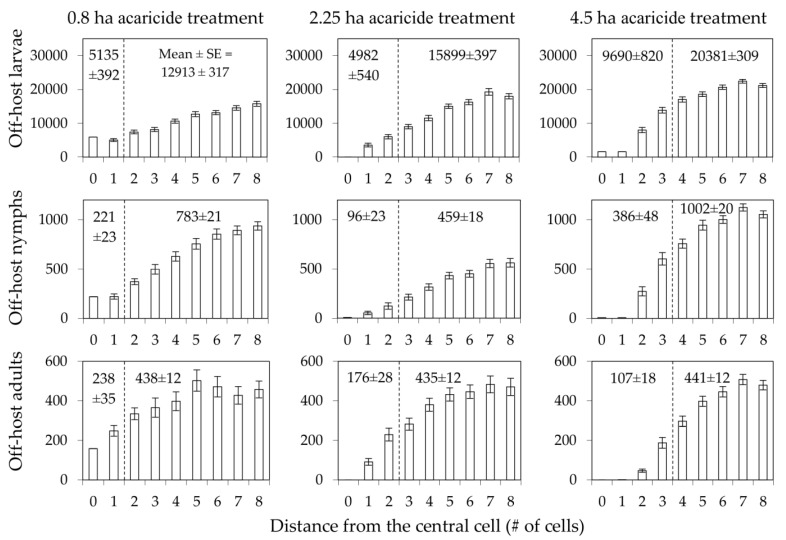
Densities of off-host ticks (larvae, nymphs, and adults; individuals/ha) at the indicated distances from the center of 4.5, 2.25, and 0.8 ha treatment areas within which acaricides were applied to medium-sized hosts. Bars represent means and standard errors of late-summer/early-fall peak densities (≈week 35 for adults and ≈week 40 for larvae and nymphs) during the 4th (last) year of simulated time.

Densities exhibited a smooth gradient across the edges of the treatment areas within which acaricides were applied to medium-sized hosts, with depressed densities extending ≈100 m outward from the edges, but with perceptible densities extending ≈60 m inward from the edges, thus reaching the center of the 0.8 ha treatment area (Figure 5). Thus, the simulated treatment areas lost their effectiveness as size was decreased to slightly less than one-half the diameter of the activity range of the targeted host.

## 4. Discussion

The interactions of ticks with a variety of hosts within heterogeneous landscapes under variable climatic conditions results in a complex set of temporal and spatial patterns that are difficult to interpret without a holistic systems perspective. The systems model developed by Wang et al. [33] produces simulation results with sufficient spatial-temporal detail to represent the potential effects of local and/or periodic tick control measures. Our adaptation of this model to examine the effects of exclosures and acaricides targeted at medium-sized hosts on lone star tick populations provides new insight into the ecology and management of tick-host systems. In particular, our simulation results indicate that off-host nymph densities decreased in response to the exclusion of, and the application of acaricides to, medium-sized hosts. These results suggest that development of tick suppression tactics targeted at medium-sized hosts may merit further consideration. The targets of most tick control programs have been small- and large-sized hosts [11,38]. Control programs focused on lone star ticks, blacklegged ticks (*Ixodes scapularis*), and American dog ticks (*Dermacentor variabilis*) have used physical exclusion of large hosts such as white-tailed deer or the use of acaricides in systems that include treatment of both livestock and deer [20,21]. The rationale is that large hosts feed the largest number of adult ticks, which lay many eggs (>8000 eggs per engorged female), which can produce large populations of larvae [39]. Large hosts also can provide blood meals to all three off-host life stages. Alternatively, providing white-footed mice (*Peromyscus leucopus*) access to acaricide-treated rodent nesting material has been effective in controlling immature blacklegged ticks, which prefer this host [13]. Our simulation results also suggest that the success of control strategies for lone star ticks may be affected markedly by time of year. For example, late summer-to-fall control of medium-sized mammalian hosts probably would produce the largest impact on tick populations. This is the time of year when mammal populations peak (at the end of their annual reproductive cycle) and share the landscape with the nymph population that has overwintered, the current year’s adult population, and current year’s nymph generation. 

Our simulations explored the potential effects of six hypothetical “best case” scenarios involving novel tick control strategies targeted at medium-sized hosts. “Best case” in the sense that we assumed that all targeted hosts were excluded from the treatment areas, that acaricides applied to targeted hosts killed all on-host ticks, and that treatments were maintained continuously for four years. Although beyond the scope of the present study, our model could be used to explore a variety of alternative scenarios in which the effectiveness of exclosures, the efficacy of acaricides, and the timing and duration of treatments are modified. Our model also could be reparametrized to represent different climatic conditions, landscape features, and host community composition.

Strategies proposed to reduce tick-related problems, which often include human health risks and large economic losses associated with reduced production of domestic livestock, invariably include tick control. Field observations collected at spatial-temporal scales allowing the identification of cause-effect connections among climatic conditions, landscape features, host community composition, and parasite life cycles do not exist. Pending the availability of such field data, simulation models such as the one we have used in the present study can facilitate a better understanding of the spatial-temporal dynamics of tick populations in response to novel control treatments under specific local conditions.

## Data Availability

The datasets generated during and/or analyzed during the current study are available from the corresponding author on reasonable request.
